# Inhaled Treprostinil Dosage in Pulmonary Hypertension Associated With Interstitial Lung Disease and Its Effects on Clinical Outcomes

**DOI:** 10.1016/j.chest.2022.09.007

**Published:** 2022-09-15

**Authors:** Steven D. Nathan, Chunqin Deng, Christopher S. King, Hilary M. DuBrock, Jean Elwing, Sudarshan Rajagopal, Franz Rischard, Sandeep Sahay, Meredith Broderick, Eric Shen, Peter Smith, Victor F. Tapson, Aaron B. Waxman

**Affiliations:** a Inova Fairfax Hospital, Fall Church, VA; b United Therapeutics Corporation, Research Triangle Park, NC; c Mayo Clinic Rochester, Rochester, MN; d University of Cincinnati College of Medicine, Cincinnati, OH; e Duke University School of Medicine, Durham, NC; f University of Arizona College of Medicine, Tuscon, AZ; g Houston Methodist Lung Center, Houston Methodist Hospital, Houston, TX; h Cedars-Sinai Medical Center, Los Angeles, CA; i Inari Medical, Irvine, CA; j Brigham and Women’s Hospital, Boston, MA

**Keywords:** interstitial lung disease, prostacyclin, pulmonary hypertension

## Abstract

**Background:**

Pulmonary hypertension (PH) complicates the course of many patients with fibrotic interstitial lung disease (ILD). Inhaled treprostinil (iTre) has been shown to improve functional ability and to delay clinical worsening in patients with PH resulting from ILD.

**Research Question:**

Do higher dosages of iTre have greater benefits in preventing clinical worsening and achieving clinical improvement?

**Study Design and Methods:**

Post hoc analysis of the INCREASE study, a 16-week double-blind, randomized, placebo-controlled trial of iTre in patients with PH resulting from ILD. Four groups were identified based on the number of breaths per session (bps; < 9 and ≥ 9 bps) of active drug or placebo attained at 4 weeks. Patients were evaluated for clinical worsening (15% decrease in 6-min walkdistance, cardiopulmonary hospitalization, lung transplantation, or death) or clinical improvement (15% increase in the six-minute walk distance with a concomitant 30% reduction in N-terminal prohormone of brain natriuretic peptide without any clinical worsening event).

**Results:**

At 4 weeks, 70 patients were at a dose of ≥ 9 bps (high-dosage group) and 79 patients were at a dose of < 9 bps (low-dosage group) in the iTre arm vs 86 patients in the high-dose group and 67 patients in the low-dose group in the placebo arm. Between weeks 4 and 16, 17.1% of patients in the high-dose treprostinil group and 22.8% in the low-dose treatment group experienced a clinical worsening event vs 33.7% and 34.3% of patients in the two placebo arms, respectively (*P* = .006). By week 16, 15.7% and 12.7% of patients in the high- and low-dose iTre groups, respectively, demonstrated clinical improvement vs 7% and 1.5% patients in the placebo arms (*P* = .003)

**Interpretation:**

Higher dosages of iTre overall show greater benefit in terms of preventing clinical worsening and achieving clinical improvement. These data support the early initiation and uptitration of therapy to a dosage of at least 9 bps four times daily in patients with PH resulting from ILD.

**Trial Registry:**

ClinicalTrials.gov; No.: NCT02630316; URL: www.clinicaltrials.gov

## Graphical Abstract

**Figure:**
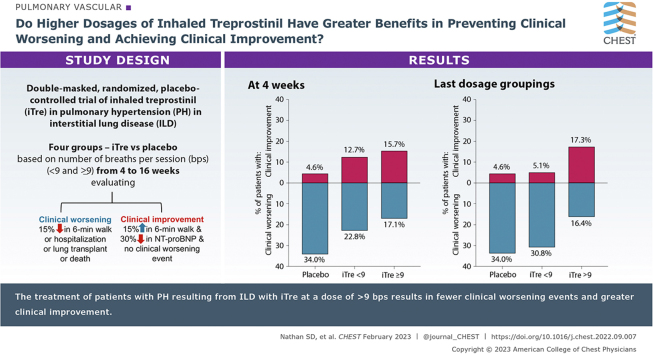

**Take-home Points**

**Study Question:** What is the impact of higher dosages of inhaled treprostinil on clinical outcomes in patients with pulmonary hypertension (PH) resulting from interstitial lung disease (ILD)?

**Results:** After 4 weeks, inhaled treprostinil (iTre) at a dose of ≥ 9 breaths per session (bps) four times daily resulted in less clinical worsening and more clinical improvement during the subsequent 3 months.

**Interpretation:** The treatment of patients with PH resulting from ILD with iTre at a dose of ≥ 9 bps results in fewer clinical worsening events and greater clinical improvement.

Pulmonary hypertension (PH) commonly complicates the course of patients with various forms of fibrotic interstitial lung disease (ILD). When it does occur, it is associated with significant functional impairment, worse quality of life, and increased mortality.^1^ Whether to treat PH associated with ILD has been a controversial issue.^1^^,^^2^ Which patients might benefit from therapy, and what defines these phenotypic patients, has been a topic of much debate. Numerous case reports and retrospective series have been reported, but few randomized clinical trials have addressed this issue.^2^ Until recently, the few randomized clinical studies have been equivocal at best, and in some cases even harmful.^3^^,^^4^

The INCREASE study, a phase 3 double-blind placebo-controlled trial of inhaled treprostinil (iTre), is the largest study to date in patients with PH resulting from ILD.^5^ This 16-week trial included patients with the diagnosis of PH resulting from ILD confirmed by right heart catheterization and CT imaging. Patient were treated with iTre or placebo initially at 3 breaths per session (bps) four times daily and titrating up to 9 to 12 bps four times daily. The study met its primary end point of change in 6-min walk distance (6MWD) at week 16. Additionally, patients receiving iTre showed a lower risk of clinical worsening than patients receiving placebo as defined by either cardiopulmonary hospitalization, 6MWD decrease of > 15% from baseline, lung transplantation, or death.

In the INCREASE study, higher doses of iTre were associated with greater improvements in 6MWD. However, it is unknown whether higher doses of iTre in patients with PH resulting from ILD are associated with other benefits such as decreased rates of clinical worsening. The goal of this post hoc analysis therefore was to evaluate whether patients achieving higher doses did indeed demonstrate better outcomes based on the end points of clinical worsening or clinical improvement.

## Study Design and Methods

Details of the INCREASE study procedures and end points have been reported previously.^5^ Patients were started on iTre or placebo at a dose of 3 bps four times daily. ITre (0.6 mg/mL) or placebo was administered by an ultrasonic, pulsed-delivery nebulizer at 6 μg/breath. The dosage was escalated by an additional 1 bps as often as every 3 days, with a target dose of 9 bps four times daily and a maximum dose of 12 bps four times daily. Investigators adjusted the dose on an individual patient basis to achieve the maximum tolerated dosage. Therefore, the earliest patients could achieve the lowest target dose of 9 bps was 21 days. To allow for sufficient time to uptitrate the dose of iTre, the week 4 dose was used to stratify patients by dosage: < 9 bps (< 54 μg) and ≥ 9 bps (≥ 54 μg). The decision to analyze patients for clinical events only after week 4 was intended to avoid any biases imposed by the early uptitration period. Four groups of patients were analyzed independently for outcomes: iTre ≥ 9 bps at 4 weeks, iTre < 9 bps at 4 weeks, placebo ≥ 9 bps at 4 weeks, and placebo < 9 bps at 4 weeks. If patients continued to increase the dosage beyond 4 weeks to ≥ 9 bps or the dosage was decreased from ≥ 9 to < 9 bps, then they were still analyzed in the original groups similar to that of an intention-to-treat analysis. Dosing of individual patients was tracked closely through a study diary and the reconciliation of used and unused study drug ampules at each study visit. Those who did not reach the week 4 study visit were not included in this analysis.

The coprimary end points of this study were the proportion of patients who experienced a clinical worsening event or who demonstrated clinical improvement between weeks 4 and 16 in each group. Clinical worsening was defined by one of four events: a 15% decrease in the 6MWD, hospitalization for cardiopulmonary cause, lung transplantation, or death. Clinical improvement was defined by a 15% increase in the 6MWD by 16 weeks accompanied by a 30% reduction in N-terminal prohormone of brain natriuretic peptide in the absence of any clinical worsening events.

### Statistical Analysis

The Cochran-Mantel-Haenszel Pearson χ ^2^ test was used to compare overall rates of clinical worsening and overall rates of clinical improvement between treatment groups adjusted for the dosage groups and for baseline 6MWD category (≤ 350 m vs > 350 m). The baseline demographics, hemodynamics, and lung function among the four groups was compared using Cochran-Mantel-Haenszel test for categorical variables and the Wilcoxon (van Elteren) test for continuous variables.

## Results

**Figure 1:**
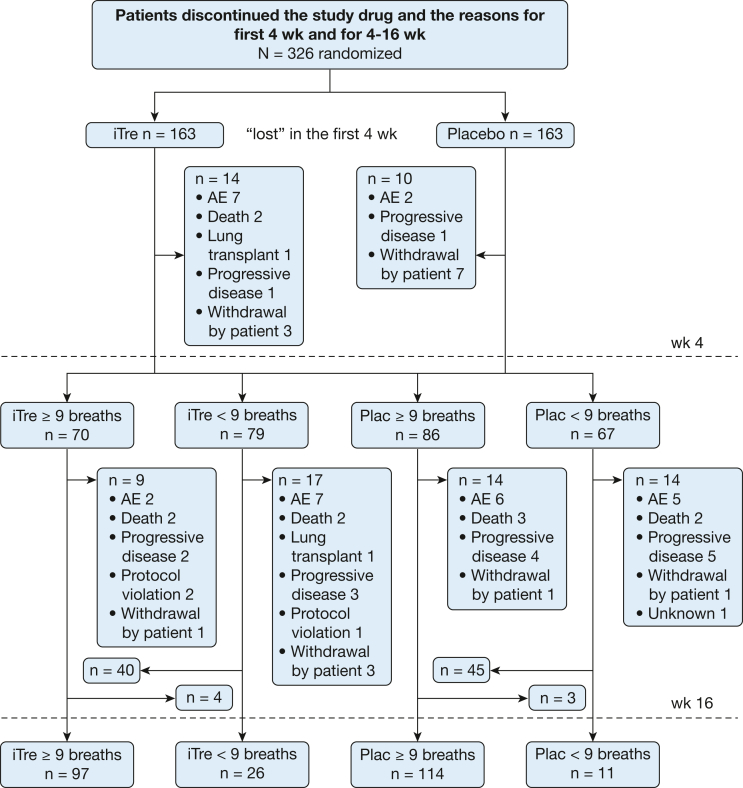
Consolidated Standards of Reporting Trials diagram depicting patient disposition based on dosage. AE = adverse event.

**Figure 2:**
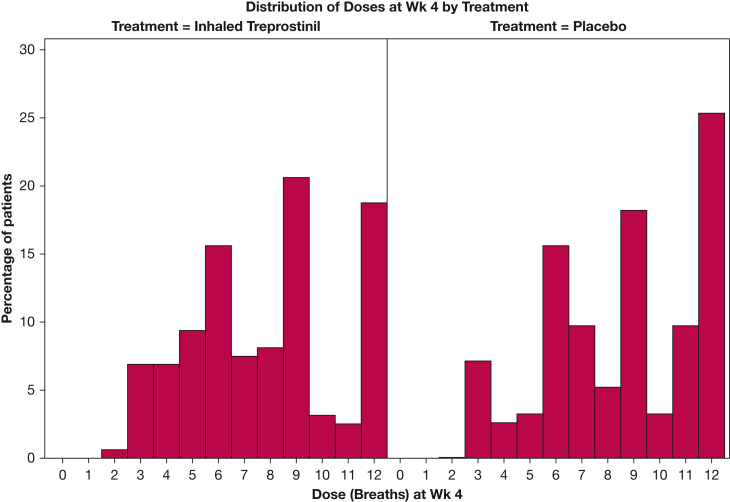
Bar graph showing the distribution of dosages at week 4.

The INCREASE study enrolled 326 patients with PH resulting from ILD who were randomized equally to receive iTre or placebo in a double-blind fashion. The baseline characteristics of the study cohort have been described previously.^3^ Figure 1 provides a Consolidated Standards of Reporting Trials diagram depicting patient disposition based on dosage. Fourteen patients in the iTre arm dropped out before week 4 vs 10 patients in the placebo arm. Of these, seven patients in the iTre group experienced a clinical worsening episode compared with two patients in the placebo arm before week 4. At 4 weeks, 149 patients were in the iTre group and 153 patients were in the placebo group. Of these, 70 patients receiving iTre had achieved a dosage of *>* 9 bps vs 86 patients in the placebo group. Seventy-nine patients receiving iTre and 67 patients receiving placebo remained at < 9 bps at 4 weeks. The median dosages in the iTre < 9 bps and ≥ 9 bps groups were 6 and 12 bps, respectively. The patients categorized into these four groups formed the primary cohort for this analysis. The demographics, hemodynamics, and lung function at baseline of the four groups at week 4 are shown in Table 1. The four groups were well balanced with regard to baseline demographics and clinical variables. Baseline values for diffusing capacity for carbon monoxide % predicted and mean pulmonary arterial pressure were higher in the iTre ≥ 9 bps group and the iTre < 9 bps group, respectively, but did not reach significance. The distribution of dosages at week 4 is shown in Figure 2.

**Table 1:** Baseline Demographics, Hemodynamics, and Lung Function of the Four Groups at the Start of the INCREASE Study

Characteristic	iTre ≥ 9 bps (≥ 54 μg; n = 70)	Placebo ≥ 9 bps (n = 86)	iTre < 9 bps (< 54 μg; n = 79)	Placebo < 9 bps (n = 67)	*P* Value^a^
Female sex	32 (45.7)	35 (40.7)	45 (57)	28 (41.8)	.287
Age, y	70 (26-89)	71 (37-85)	68 (31-88)	70 (36-85)	.224
ILD type					.648
IIP	32 (45.7)	41 (47.7)	29 (36.7)	36 (53.7)	
Chronic HP	5 (7.1)	6 (7)	4 (5)	1 (1.5)	
CPFE	16 (22.9)	25 (29.1)	23 (29.1)	13 (19.4)	
CTD-related ILD	15 (21.4)	13 (15.1)	22 (27.9)	17 (25.4)	
Other	2 (2.9)	1 (1.2)	1 (1.3)	0	
Oxygen, L/min	3 (0-8)	2 (0-8)	3 (0-10)	2 (0-6)	.346
FVC % predicted	60 (27-130)	61 (25-130)	60 (24-112)	60.5 (20-134)	.600
FEV_1_ % predicted	63 (23-120)	62 (25-117)	62.5 (27-103)	66 (22-145)	.796
Dlco % predicted	30 (5-54)	25 (8-71)	26.5 (7-86)	27 (1-86)	.173
6MWD, m	268.5 (100-533)	249 (30-491)	232 (101-538)	285 (105-505)	.147
NT-proBNP, pg/mL	488 (10-21,942)	476 (41,16297)	551 (10-12,848)	321 (23-14,331)	.568
mPAP, mm Hg	34.5 (25-66)	35 (25-61)	37 (25-74)	33 (25-57)	.081
PVR, Wood units	5.4 (3.1-16.1)	5.3 (3-17.6)	5.55 (3.2-18)	4.7 (3.1-11.2)	.198

Data are presented as No. (%) or median (range), unless otherwise indicated. 6MWD = 6-min walk distance; bps = breaths per session; CPFE = combined pulmonary fibrosis and emphysema; CTD = connective tissue disease; Dlco = diffusing capacity for carbon monoxide; HP = hypersensitivity pneumonitis; IIP = idiopathic interstitial pneumonia; ILD = interstitial lung disease; IPF = idiopathic pulmonary fibrosis; iTre = inhaled treprostinil; mPAP = mean pulmonary artery pressure; NT-proBNP = N-terminal prohormone of brain natriuretic peptide; PVR = pulmonary vascular resistance.

a Cochran-Mantel-Haenszel test for categorical variables and stratified Wilcoxon (van Elteren) test for continuous variables.

Forty patients in the iTre < 9 bps group uptitrated to iTre ≥ 9 bps between weeks 4 and 16, whereas four patients downtitrated from iTre ≥ 9 bps to iTre < 9 during this period. For the placebo arm, 45 and 3 patients, respectively, switched groups (Fig 1). At week 16, 97 patients completed the study at iTre ≥ 9 bps four times daily and 26 patients completed the study at iTre < 9 bps, whereas in the placebo groups, 114 patients completed the study at placebo at ≥ 9 bps and 11 patients completed the study at placebo < 9 bps. Figure 1 shows the disposition of the patients between weeks 4 and 16.

**Figure 3:**
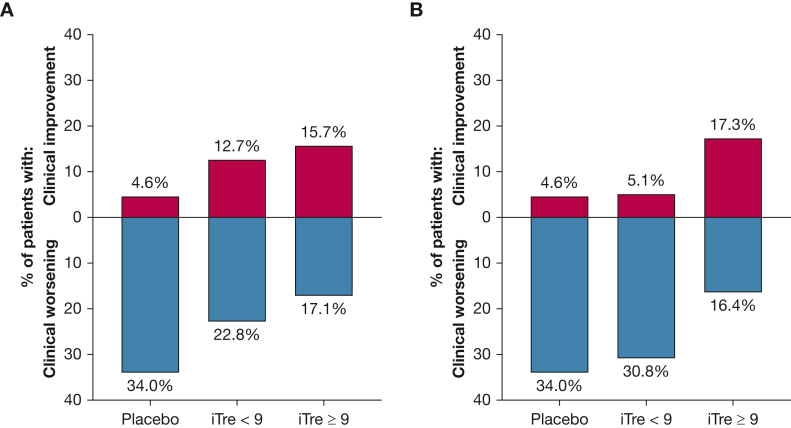
Bar graphs showing clinical improvement and clinical worsening in patients who were receiving iTre ≥ 9 bps four times daily vs iTre < 9 bps four times daily vs the two placebo groups (combined for this figure): based on groupings at week 4 (A) and based on the last dosage groupings (B). iTre = inhaled treprostinil.

Twelve of 70 patients (17.1%) in the iTre ≥ 9 bps group experienced a clinical worsening event between weeks 4 and 16 vs 18 of 79 patients (22.8%) in the iTre < 9 bps group vs 29 of 86 patients (33.7%) and 23 of 67 patients (34.3%) in the placebo ≥ 9 bps and < 9 bps groups, respectively (*P* = .006). On the other end of the spectrum, eleven of 70 patients (15.7%) and ten of 79 patients (12.7%) in the high- and low-dosage iTre groups experienced clinical improvement by week 16 vs 6 of 86 patients (7%) and 1 of 67 patients (1.5%) in the placebo *>* 9 bps and < 9 bps groups, respectively (*P* = .003) (Table 2, Fig 3A).

**Table 2:** Clinical Worsening and Clinical Improvement in the Four Groups of Patients (Defined at Week 4) at Week 16

Variable	iTre *>* 9 bps (n = 70)	Placebo *>* 9 bps (n = 86)	iTre < 9 bps (n = 79)	Placebo < 9 bps (n = 67)	*P* Value^a^
Clinical worsening	12 (17.1)	29 (33.7)	18 (22.8)	23 (34.3)	.0031
Hospitalization for cardiopulmonary cause	8 (11.4)	14 (16.3)	7 (8.9)	8 (11.9)	
≥ 15% decrease in 6MWD	3 (4.3)	14 (16.3)	8 (10.1)	12 (17.9)	
Death	1 (1.4)	1 (1.2)	2 (2.5)	3 (4.5)	
Lung transplantation	0	0	1 (1.3)	0	
Clinical improvement^b^	11 (15.7)	6 (7.0)	10 (12.7)	1 (1.5)	.0028

Data are presented as No. (%), unless otherwise indicated. 6MWD = 6-min walk distance; bps = breaths per session; iTre = inhaled treprostinil.

a Cochran-Mantel-Haenszel test adjusted for baseline 6MWD category.

b Defined by a 15% increase in 6MWD accompanied by a 30% decrease in N-terminal prohormone of brain natriuretic peptide in the absence of any clinical worsening.

As a sensitivity analysis, we analyzed patients for any clinical worsening event or improvement grouped by last dosage received. For this analysis, we also included only patients who made it to week 4 for consistency with the primary analysis (Table 3). The results of this were very similar, with fewer patients in the iTre *>* 9 bps group showing clinical worsening (16.4%) compared with the other three groups (iTre < 9 bps, 30.8%; placebo ≥ 9 bps, 32.3%; placebo < 9 bps, 42.3%). In addition, more patients in the iTre > 9 bps group achieved clinical improvement (17.3%) compared with the other three groups (5.1%, 4.7%, and 3.8%, respectively) (Table 3, Fig 3B).

**Table 3:** Clinical Worsening and Clinical Improvement After Week 4 by Groups Defined by the Last Dosage Received

Variable	iTre ≥ 9 bps (≥ 54 μg; n = 110)	Placebo ≥ 9 bps (n = 127)	iTre < 9 bps (< 54 μg; n = 39)	Placebo < 9 bps (n = 26)	*P* Value^a^
Clinical worsening	18 (16.4)	41 (32.3)	12 (30.8)	11 (42.3)	.0031
Hospitalization for cardiopulmonary cause	10 (9.1)	17 (13.4)	5 (12.8)	5 (19.2)	
≥ 15% decrease in 6MWD	6 (5.5)	23 (18.1)	5 (12.8)	3 (11.5)	
Death	2 (1.8)	1 (0.8)	1 (2.6)	3 (11.5)	
Lung transplantation	0	0	1 (2.6)	0	
Clinical improvement^b^	19 (17.3)	6 (4.7)	2 (5.1)	1 (3.8)	.0025

Data are presented as No. (%), unless otherwise indicated. 6MWD = 6-min walk distance; bps = breaths per session; iTre = inhaled treprostinil.

a Cochran-Mantel-Haenszel test adjusted for baseline 6MWD category.

b Defined by a 15% increase in 6MWD accompanied by a 30% decrease in N-terminal prohormone of brain natriuretic peptide in the absence of any clinical worsening.

## Discussion

This post hoc analysis of the INCREASE trial provides evidence of a higher likelihood for clinical benefit using iTre, especially at higher dosages, when compared with placebo in patients with PH resulting from ILD. Our analysis evaluated clinical change 4 weeks after study entry to allow sufficient time for drug uptitration. The benefit was seen on both ends of the clinical outcome spectrum, with both lower rates of clinical worsening and higher rates of clinical improvement experienced in the iTre group compared with the placebo arm during the 16-week study. These benefits also appeared most apparent with higher dosages of iTre. Approximately three-quarters of patients were able to achieve a dosage of ≥ 9 bps four times daily, suggesting that titrating to higher dosages of iTre is feasible in most patients over a relatively short period with appropriate adverse event management.

This analysis further reinforces that iTre prevents clinical worsening and in addition demonstrates that iTre is associated with significantly more patients experiencing clinical improvement when compared with those receiving placebo. Furthermore, our analysis suggests that higher-dosage iTre is more effective than lower-dosage in achieving these outcomes. The results from this analysis in patients with PH resulting from ILD are consistent with previous reports in World Health Organization group 1 pulmonary arterial hypertension (PAH), which suggest that higher doses of iTre have beneficial effects on survival and delayed time to parenteral therapy.^6^ However, our analysis differs from prior studies in that we evaluated patients for both clinical worsening and clinical improvement. One way to combine the beneficial effect of iTre is to evaluate the number needed to treat either to prevent one clinical worsening event or to enable one clinical improvement event. This is calculated by the inverse of the absolute difference between the treatment and placebo arms for clinical worsening and clinical improvement. For example, the absolute risk reduction for the iTre ≥ 9 bps group vs the placebo group for clinical worsening was 16.6% at 16 weeks (33.7% minus 17.1%), whereas the difference in clinical improvement at 16 weeks was 8.7% (15.7% minus 7%). Therefore, the combined absolute difference in outcomes was 25%, which translates to a number needed to treat of four (1 divided by 0.25) either to prevent one clinical worsening event or to enable clinical improvement by week 16.

To our knowledge, clinical improvement has not been evaluated previously in any ILD study and is rarely sought in PAH clinical trials. The REPLACE study evaluated the switching of phosphodiesterase 5 inhibitor therapy to riociguat with clinical improvement as the primary end point.^7^ This was defined as an absence of clinical worsening accompanied by at least two of the following three variables: 6MWD increase of ≥ 10%, World Health Organization functional class 1 or 2, or N-terminal prohormone of brain natriuretic peptide reduction of ≥ 30%. Our definition of clinical improvement was similar; we used a higher 6MWT threshold, but did not include the World Health Organization functional class because this was not captured in the INCREASE study. We believe that any future PH resulting from ILD studies should have clinical improvement as an end point since this is an important patient-centric measurement. The ability to offer patients a therapy that may result in improvement rather than one that will only delay worsening likely will resonate better with patients and may facilitate improved compliance. This also can be regarded as a more proactive treatment strategy, rather than only “playing defense” by preventing worsening. A similar mindset shift has taken place in the treatment of PAH, in which the goal of therapy now is to improve patients’ overall risk profiles, rather than just preventing patients’ conditions from worsening.^8^

Because the concept of clinical improvement in PH resulting from ILD largely is unexplored, what should be regarded as constituting improvement is open to debate. We chose a 15% increase in the 6MWD, which for most patients would be in the range of the minimally important difference that has been estimated to be anywhere from 22 to 50 m in patients with IPF, the most common of the ILDs.^9^^,^^10^ This also is similar to the minimally important difference in PAH.^11^ However, no evaluation of the 6MWD minimally important difference in PH resulting from ILD has been undertaken, which we assume would be within a similar range as PAH. To further validate this 15% increase as meaningful, we imposed the additional prerequisite of a 30% decrease in the N-terminal prohormone of brain natriuretic peptide. We believe that this objective assessment of reduced right ventricular strain to be a valuable adjunct in confirming that the increase in the 6MWD indeed was the result of a treatment effect, rather than a chance finding. In addition, to qualify as improved, patients could not experience any worsening events, specifically any cardiopulmonary hospitalizations, because this is the only possible worsening event after which patients could still show improvement.

The decision to analyze patients for clinical events only after week 4 was to avoid any biases imposed by the early uptitration period. Specifically, if we had analyzed patients for events in the first 4 weeks, then the bias would have been toward more clinical worsening events in the low-dosage group, because all the patients were in the uptitration phase during this period. However, the fact that a sizable group of patients did show clinical worsening during this early phase of the study does underscore the need for the early implementation of therapy. During the 4- to 16-week period, 29.5% of patients (44 of 149) switched groups. However, which group should their clinical worsening be attributed to if they switched over and then experienced an event shortly thereafter? For this reason, we believe that the best analysis was to adopt the equivalent of an intention-to-treat approach based on the groupings at week 4. Given this limitation to our analysis, we performed a sensitivity analysis of clinical events of the same four groupings based on the last dosage received. This analysis demonstrated very similar results and reinforces the robustness of our primary analysis. Another option would have been to perform an analysis based on the total exposure to iTre by using the cumulative dose of iTre for each patient through the 16-week trial and then to determine the median cumulative dose to define the cutoff for the high- and low-dosage subgroups. However, in a dose-response analysis using cumulative dose, any patient who discontinued the study early or died during the study invariably would be assigned to the low-dose group. This would skew the results favorably to the high-dose group, and therefore, we do not believe it would paint an accurate picture of the true dose-response relationship for iTre.

Limitations of this analysis include the unavoidable issue of patients crossing groups between 4 and 16 weeks and the inherent difficulty in clinical event attribution. However, we believe that we adequately addressed this between the primary and secondary analyses, both of which showed very similar results. The relatively short duration of the study is another limitation; it is certainly likely that we would have observed more events with a longer duration of follow-up. We chose to start the analysis at 4 weeks because this allowed adequate time for most patients to uptitrate and also coincided with a mandated study visit. As discussed, any clinical worsening events during this initial period would have been ascribed to the lower dosage group, which would have introduced an inherent bias favoring the higher-dosage group. The fact that a number of clinical worsening events occurred during this early period of dose optimization supports the concept of high vigilance and screening for interceding pulmonary hypertension in patients with ILD with the goal of early drug implementation. We also cannot rule out that tolerability of higher dosages could be a surrogate for disease severity. For instance, patients who are unable to titrate up may have clinical features putting them at higher risk of poor outcomes. Specifically, the iTre < 9 bps group showed a lower baseline 6MWD compared with the iTre ≥ 9 bps group. This provides further support for the earlier initiation of therapy. Finally, clinical worsening events were not adjudicated centrally, but the components of this composite end point are mostly objective.

## Interpretation

This post hoc analysis demonstrated that a higher doses of iTre is associated with an overall greater benefit in patients with PH resulting from ILD, not only in comparison with placebo, but also in comparison with lower doses of iTre. This benefit is magnified when one considers both the prevention of clinical worsening and the attainment of clinical improvement. These results support the importance of early initiation and uptitration of iTre therapy to ≥ 9 bps four times daily. Our analysis lays the foundation for an improvement end point in clinical trials of PH resulting from ILD, thereby raising the bar to meet what patients with PH resulting from ILD hope for and deserve.

## Funding/Support

The INCREASE study was sponsored by United Therapeutics Corporation but this manuscript was not funded by any entity. S. R. is supported by the National Heart, Lung, and Blood Institute [Grant R01HL153872], the National Institute of General Medical Sciences [Grant R01GM122798], and the American Heart Association [Grant TPA34880033]. F. R. is supported by the National Institutes of Health and the National Heart, Lung, and Blood Institute. V. F. T. is supported by the National Institutes of Health.

## Financial/Nonfinancial Disclosures

The authors have reported to *CHEST* the following: S. D. N. is a consultant and on the speakers bureau for United Therapeutics, is on the speaker bureau for Bayer and Boehringer-Ingelheim, and is a consultant for Boehringer-Ingelheim, Roche, Merck, Bellerophon, and Third Pole. C. D., M. B., E. S., and P. S. are employees of United Therapeutics. C. S. K. is on the speaker and advisory boards for Boehringer-Ingelheim, Actelion, and United Therapeutics. H. M. D. is a consultant for Janssen Pharmaceuticals, has received grant funding from Bayer, and has served on advisory boards for United Therapeutics and Janssen Pharmaceuticals. J. E. has received research support and grants from Janssen, United Therapeutics, Liquidia, Phase Bio, Gossamer Bio, Bayer, Acceleron, Altavant, and Aerovate and advises and consults for Actelion/Janssen, United Therapeutics, Acceleron, Liquidia, Altavant, Aerovate, Bayer, and Gossamer Bio. S. R. has consulted for Apie Therapeutics, Altavant, GossamerBio, Insmed, Janssen, Liquidia, Polarean, and United Therapeutics, has received funding support from Janssen and United Therapeutics, and is a patent-holder on US patent 62/673,175 entitled “Dynamic 129Xe Gas Exchange Spectroscopy” that is licensed to Polarean. F. R. is a consultant for Acceleron and United Therapeutics and is on a steering committee for Acceleron, Ismed, United Therapeutics, Bayer, Acceleron, Janssen, and AADI. S. S. has received funds from United Therapeutics (industry-initiated research, consulting, and speaking [promotional and nonpromotional]), from Liquidia Technologies (consulting, steering committee), from Gossamer Bio (consulting), from Bayer (consulting, advisory committee, speaking [promotional and nonpromotional]), from GlaxoSmithKline (adjudication committee), and from Johnson & Johnson (speaking [promotional and nonpromotional]). V. F. T. is an employee of Inari Medical and has received research support from Bayer, Boston Scientific Corporation, Daiichi, Genentech, and Janssen; he is on steering committees for Bayer, Bi02 Medical, Penumbra, Daiichi, Thrombolex, and United Therapeutics; is a consultant for Janssen and Bi02; and has received speaking honoraria from Janssen. A. B. W. is an investigator, study investigator, and steering committee chair for United Therapeutics; an investigator and steering committee member for Acceleron; is a principle investigator for Aria-CV; has received an investigator-initiated grant from Janssen R&D; R01HL158077 Co-I; R01HL160025 Co-I; and is a co-principle investigator for PVDomics.
